# A Case of Lupus Treated with Oils

**Published:** 1901-09

**Authors:** N. F. Howard

**Affiliations:** Dahlonega, Ga.


### A CASE OF LUPUS TREATED WITH OILS.

By N. F. HOWARD, M.D.,

Dahlonega, Ga.

I was informed some mouths ago that an advertisement was published from some medical practitioner, Dr. Bye, of Indiana, making the announcement that he was able to "cure," or relieve all skin diseases, including lupus, cancer, etc., by the use of vegetable oils. How true the statement of the fact may be, I do not know, but what a blessing it would be, if a remedy could be discovered which would deliver the human race from the dreadful and mortal disease—cancer, and restore the patient to a condition of health.

If THE ATLANTA JOURNAL-RECORD OF MEDICINE will give me a place for publication, I will give the profession my experience with the oils as a medicinal agent in the treatment of skin diseases, as well as lupus, sometimes called "cancer."

Three years ago I met a friend in the country, who had suffered with lupus and had been relieved. I will state here that quite a number of that family had suffered with the same disease. Some of them had large ulcers on the face and nose, which they called "eating cancers."

A friend of mine told me he had the recipe of the medicine that cured his face, and it embraced several oils; the recipe of which he gave me. I told him I had treated such cases with the charcoal from the white bloomed sorrel, but at this time I was treating a case with bismuth, white vaseline and English resin made into an ointment. He said: "The recipe is as follows: Equal quantities of castor oil, sweet oil, linseed oil, British oil, and gum camphor; mix by gentle heat. Apply this to the parts affected two or three times a day."

I decided to give this preparation a trial in my case of lupus, which was one that often appeared on the nose, for a good many years past; but before it had advanced far had been relieved from time to time with the charcoal made from sorrel. At this time it had seated itself on the face, near the nose. I applied the oil preparation night and morning, and during the day a plaster of the bismuth salve. In the course of two or three weeks there was quite a change; the lupus became tender and suddenly opened as an abscess, and discharged quite an amount of dark, sticky, very offensive matter, leaving quite a little cavity. In three days all the matter had been discharged. The cavity was kept filled with lint and the oil for a short time. The ulcer soon filled up from the bottom and healed up nicely. There has been no return of the disease these three years. We do not know which aided most in the relief of this case, the oils or the salve. The following is the formula of the salve: Four drachms bismuth, four drachms white vaseline, one drachm English resin. Mix and combine by moderate heat.

I am informed that there is quite an interest manifested by the medical profession at this time, and investigations are being made into the medicinal virtues of the oils in their use and treatment of skin diseases. I feel justified, therefore, in bringing into notice to the medical profession any information that may come to my knowledge in regard to the potency of any medicine that might prove beneficial and helpful to suffering humanity. I do not think it would be right to let one man in Indianapolis, or any other place, know all this and the others remain in ignorance of it.

I am at present treating a few cases of eczema and skin diseases with this oil preparation and solution of Epsom salts combined. I hope soon to be able to report successful results on these cases.

---

### CRAMPS OF THE LEGS.

Dr. John McDonald, after discussing the causation of cramps, their relation to the valveless condition of the inferior vena cava, and consequent great hydraulic pressure, to constipation with its pressure on the iliac veins, and to the gouty diathesis leading to the deposit of urates in the muscles surrounding the congested veins of the legs, says that in the remedial treatment of cramps, the attention should be directed mainly toward (1) the relief of constipation; (2) the removal of the uric acid toxin; and (3) the establishment of a better nutrition.

It is obvious that for this purpose an effective cholagogue agent is of the first importance to stimulate cellular action of the liver; increase its normal secretions, and initiate peristalsis; and that, combined with an appropriate uric acid solvent, the circulation of the blood may be quickened, while at the same time its sub-alkalinity may be utilized and oxidation increased by the removal of the toxin mainly responsible for the abnormal condition.

A more active interchange having thus been established between blood and tissue, the former being better enabled to perform its function of removing poisonous waste, the nutrition of the latter becomes improved, and the third indication is fulfilled. The author records a case of obstinate cramps treated successfully on these lines.—*Northwestern Lancet.*

